# Sustainably Adjusting the Up-Conversion White-Emitting Luminescence Properties of GdAlO_3_: Er^3+^/Yb^3+^/Tm^3+^ Phosphors

**DOI:** 10.3389/fchem.2020.00788

**Published:** 2020-09-29

**Authors:** Taoli Deng, Xianbang Jiang, Qiuyun Zhang

**Affiliations:** School of Chemistry and Chemical Engineering, Anshun University, Anshun, China

**Keywords:** sustainable chemistry, white-emitting, heteroatom-containing compounds, laser power, phosphors

## Abstract

Doping heteroatom in phosphor can effectively improve luminescent properties, which has attracted great attention recently. GdAlO3 phosphors (GAP) doped with Er^3+^/Yb^3+^/Tm^3+^ were prepared via the co-precipitation method. Upon 980 nm excitation, strong blue, green, and red up-conversion (UC) emissions centered at 476, 524, 546, and 659 nm were observed, which could be successfully combined to form pure white light. It was found that changing the doping concentration of Er^3+^ and Yb^3+^ ions, the calcination temperature of the precursor, the laser power of the excitation light source, and doping Li+ could systematically adjust red/green/blue colors of GdAlO3:Er3+/Yb3+/Tm3+ phosphors to optimize the white emitting luminescence. When the Er3+ doping concentration of the phosphors increased, each color distribution successfully moved, making the maximum shift of the CIE coordinate. Finally, the influence of each factor on adjusting the UC white light performance and its mechanism were explored.

## Introduction

Recently, the development of green and sustainable approaches have become a particularly important theme. The use of white light-emitting diodes (WLEDs) as a promising general illumination source in lighting and display applications has attracted great attention (Du et al., 2018; Li et al., 2018; Liu et al., 2019). There are two alternative approaches to WLEDs assembly now. The first way is to mix the red, green, and blue monochromatic light sources together to modulate white light directly. Another way is to convert the ultraviolet, blue, or infrared light sources into a combination of red, green, and blue emissions by using phosphors (DiMaio et al., 2006; Liu M. et al., 2007; Liu X. M. et al., 2007). At present, the strategy widely used in producing white light is to combine a blue LED chip with a YAG: Ce yellow phosphor (Justel et al., 1998). However, the blue light LED has low luminescence efficiency, and the device color is changed by a combination of the working temperature, voltage, and the phosphor coating thickness, which makes the white light emission unstable. Meanwhile, the lack of red light components results in a white light with both a high color temperature and a poor color rending index.

As far as we know, up-conversion photoluminescence (UCPL) can convert long wave light into short wave light and the white light obtained by UCPL can reduce the photo degradation process caused by high energy photons compared with down conversion luminescence excited by short wavelengths (Leleckaite and Kareiva, 2004; Milliez et al., 2006; Chung et al., 2012). At the same time, infrared light was used as the excitation source which had a very low cost and was easy to obtain. It is reported that UC white light can be combined by doping rare earth ions Er^3+^/Ho^3+^ emitting red and green light and Tm^3+^ emitting blue light in fluoride under 980 nm excitation (Sivakumar et al., 2005; Wang and Liu, 2008; Chen et al., 2017). While the large-scale application and industrial production of fluoride phosphors are limited by the low stability, being unfriendly to environment, and harsh conditions in the process of synthesis, it is important to find some suitable matrix materials in the UC process to obtain white light. In recent years, lots of oxides with good chemical stability, mild synthesis conditions, that are eco-friendly, and have low phonon energy are being used as the host material to obtain UC white light which has attracted researchers' attention (Rai et al., 2013; Chen et al., 2017; Annadurai et al., 2018). The GdAlO_3_ system has a orthogonal perovskite crystal structure with a Pbnm space group. The density of GdAlO_3_ is 7.437g.cm^−3^, and the phonon energy is 670 cm^−1^, which is good for UCPL (Deng and Jiang, 2018).

In our previous research on GAP, it was found that the ratio of the red to green emissions intensity can be modified after changing the Er^3+^/Yb^3+^ doping concentration and laser power. Apart from this, the particle size and the content of impurity groups adsorbed on the surface of the GAP phosphors calcined at different temperatures will also affect the intensity and proportion of red/green emissions in UCPL (Deng et al., 2014a,b). In this paper, Yb^3+^ was used to sensitize Er^3+^, Tm^3+^ in GdAlO_3_ to obtain the UC white light, and the doping concentration of Er^3+^ and Yb^3+^ ions, the calcination temperature of the precursor, the laser power of the excitation light source, and Li^+^ doping were changed to adjust the intensity and relative proportion of red, green, and blue emissions. Then the influence of each factor on UCPL performance and its mechanism were explored, which can provide guidance for the UC white light process by systematically adjusting the red /green/ blue colors.

## Experimental

The Gd_(1−x−y−z)_Er_x_Yb_y_Tm_0.01_Li_z_AlO_3_ (*x* = 0.004, 0.006, 0.008; *y* = 0.10, 0.12, 0.14, 0.16; *z* = 0, 0.02) precursors were prepared by a co-precipitation method. Firstly, stoichiometric amounts of starting rare earth (RE) oxides Gd_2_O_3_ (99.99%), Yb_2_O_3_ (99.99%), Er_2_O_3_ (99.99%), Tm_2_O_3_ (99.99%), and Li_2_CO_3_ (99.9%) were dissolved in HNO_3_ aqueous solution with the molar ratio of RE^3+^ to ${\mathrm{NO}}_{3}^{-}$ being 1:3. Then a required amount of Al(NO_3_)_3_·9H_2_O and ethanol aqueous solution were added sequentially under vigorous stirring until the homogenous solution A was formed. The beaker containing the homogenous solution A was placed in a water bath at 45°C. The 1 mol·L^−1^ NH_4_HCO_3_ aqueous solution was added into solution A at a rate of 2 mL min^−1^ with stirring. After completion of precipitation, the agitator was turned off and the precipitate was ripened at room temperature for 10 h. After ripening and filtration, the precipitate was washed with deionized water three times and ethanol two times, then dried at 120°C for 12 h. Finally, the precursor powders were calcined at different temperatures for 6 h. The crystalline GdAlO_3_:Er^3+^/Yb^3+^/Tm^3+^ phosphors were finally obtained.

The x-ray diffraction patterns of the phosphors were tested by a Bruker D8 advance diffractometer with Cu Kα radiation (λ = 0.154056 nm) operated at 30 mA and 40 kV. The UCPL spectra of the phosphors were recorded using an Ocean Optics PlasCalc-2000-UV-VIS-NIR plasma monitor control system and the exciting source was a MDL-H-980 980 nm infrared laser.

## Results and Discussion

**Figure 1:**
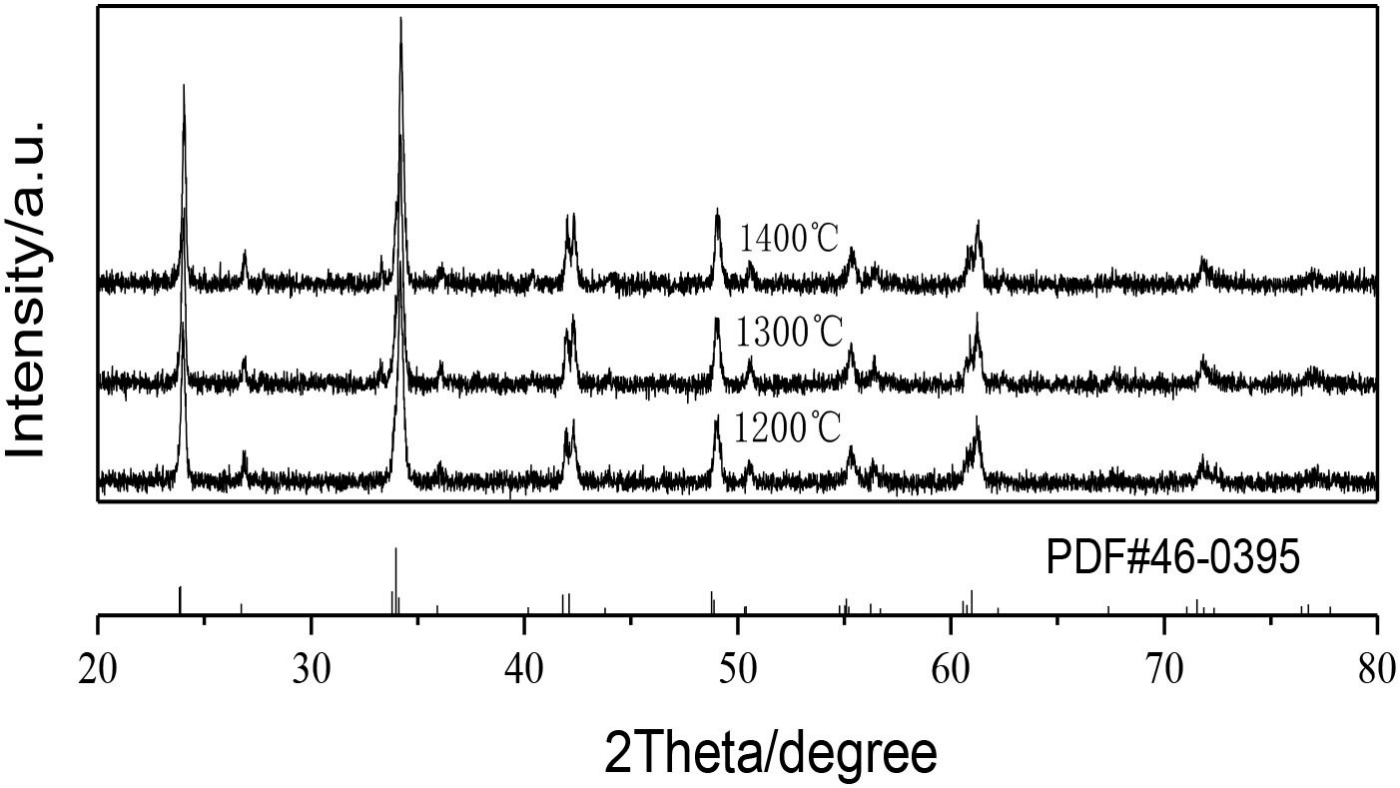
XRD patterns of the GAP phosphors prepared by calcining the precipitate at different temperatures.

Figure 1 shows the XRD patterns of the GAP phosphors prepared by calcining the precipitate at different temperatures. In the figure all the XRD diffraction peaks obtained at 1,200, 1,300, and 1,400°C can match the standard GdAlO_3_ card (PDF#46-0395) with no impurity phase being detected (Deng and Jiang, 2018), and the GdAlO_3_ host material can exist quite stably with calcination temperature from 1,200 to 1,400°C. The average sizes of the crystallites calcined at different temperatures are estimated using Scherrer's equation (Shannon, 1976):

$$\begin{array}{l} D=0.89\lambda /\beta \cos\theta \end{array}$$

where D is the average crystallite size, λ is the wavelength of the Cu Kα line, β is the full-width at half maximum in radians, and θ is the Bragg angle.

The strongest peak of the phosphors by calcining the precipitate at 1,200, 1,300, and 1,400°C, respectively, are at 34.18° (β = 0.00541), 34.20° (β = 0.00436), 34.22° (β = 0.00401) and using the procedure, the prepared phosphor particles by calcining the precipitate at 1,200, 1,300, and 1,400°C had the average crystallite sizes of 30.63 nm, 38.01 nm, 41.33 nm, which showed that a higher calcination temperature resulted in larger sized phosphor particles.

### The Effect of Er^3+^/Yb^3+^ Doping Concentration on the Tunable UC White Emissions

**Figure 2:**
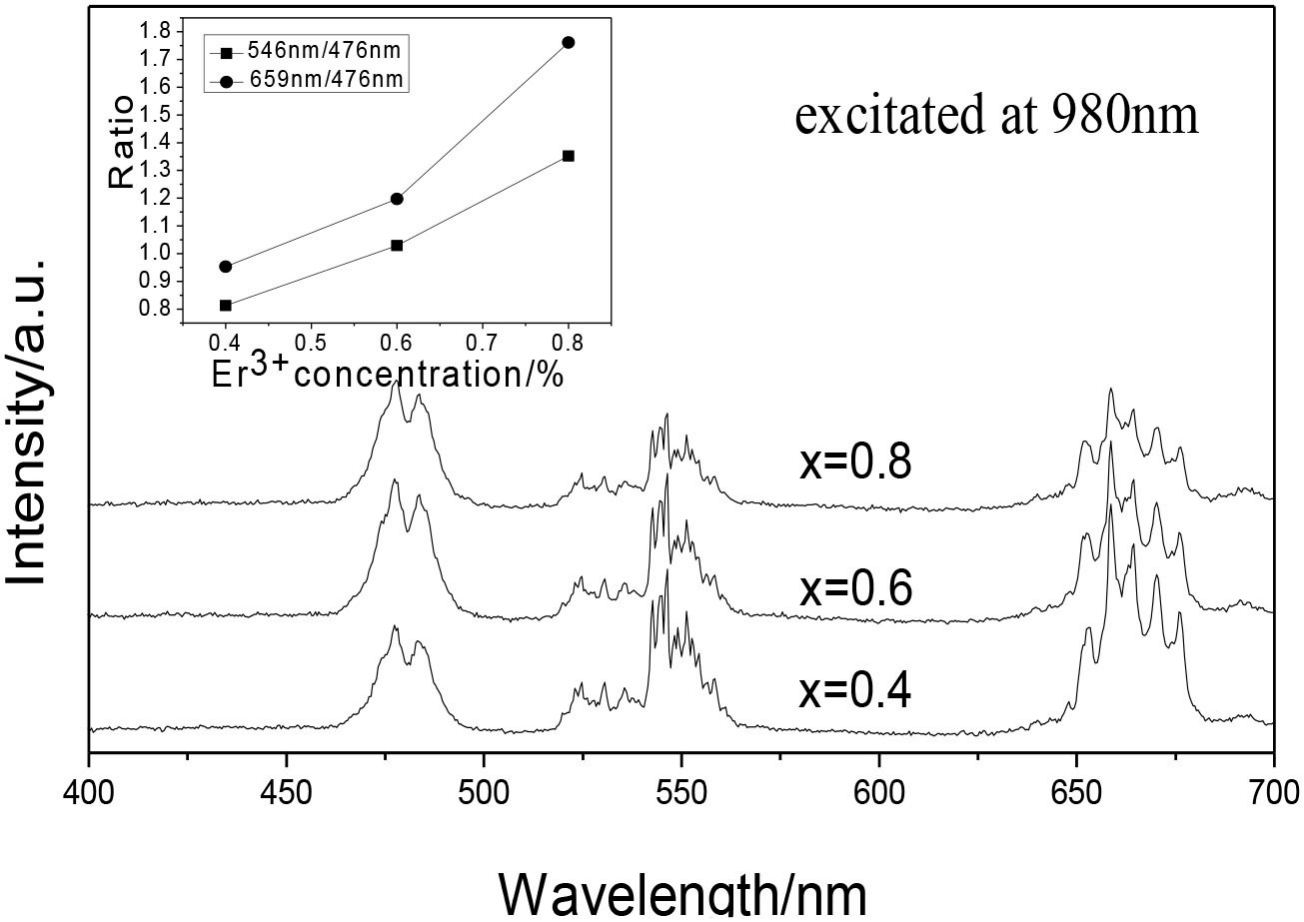
UCPL spectra of the GdAlO_3_: x%Er^3+^, 10%Yb^3+^, 1%Tm^3+^ phosphors (inset is the dependence of the 546 nm/476 nm and 659 nm/476 nm intensity ratio on Er^3+^ concentration).

Figure 2 shows the UCPL spectra of the GdAlO_3_:x%Er^3+^,10%Yb^3+^,1%Tm^3+^ (*x* = 0.4, 0.6, 0.8) phosphors and the dependence of the 546 nm/476 nm and 659 nm/476 nm intensity ratio on Er^3+^ concentration. Under excitation of 980 nm, all of the GdAlO_3_:x%Er^3+^,10%Yb^3+^,1%Tm^3+^ (*x* = 0.4, 0.6, 0.8) phosphors appeared to have four main emission peaks at 476 nm (blue), 524 nm, 546 nm (green), and 659 nm (red). The blue emission peak at 476 nm belongs to the Tm^3+^ (^1^G_4_ → ^3^H_6_) transition and the green emission peaks can be assigned to Er^3+^ (^2^H_11/2_ → ^4^I_15/2_, ^4^S_3/2_ → ^4^I_15/2_) transitions, and the red emission peak can be ascribed to the Er^3+^ (^4^F_9/2_ → ^4^I_15/2_) transition (Tamrakar et al., 2016; Cao et al., 2018). It was found that different doping concentrations of Er^3+^ does not produce a change in shape or location of the emission peaks. It can be seen from the inner illustration that the ratios of red to blue emission and green to blue emission intensity are improved at different degrees with the Er^3+^ doping concentration increase, so as to change each color distribution successfully.

**Figure 3:**
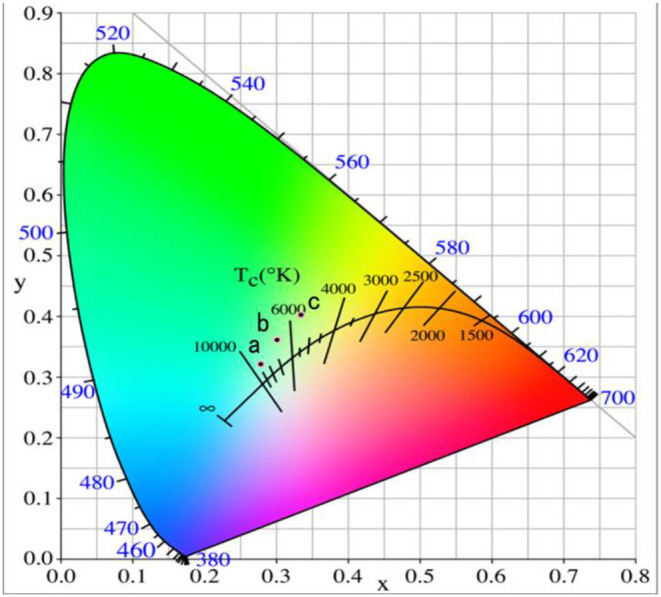
Chromaticity coordinate of the GdAlO_3_: x%Er^3+^, 10%Yb^3+^, 1%Tm^3+^ phosphors under 980 nm excitation (a: *x* = 0.4, b: *x* = 0.6, c: *x* = 0.8).

Figure 3 represents the chromaticity coordinate CIE diagram of the phosphors with different Er^3+^ doping concentrations. *When the* Er^3+^ *doping concentration was 0.04, the CIE of phosphor* GdAlO_3_:4%Er^3+^,10%Yb^3+^,1%Tm^3+^ *is (0.2787, 0.3213) shown in point a, and the CIE changed to (0.3015, 0.3609) when the* Er^3+^ *doping concentration added was 0.06, shown in point b. Finally, when the* Er^3+^ *doping concentration was 0.08, the CIE reached (0.3349, 0.4031), shown in point c. CIE significantly moves to the red and green direction, and* all the CIE of the GdAlO_3_:x%Er^3+^,10%Yb^3+^,1%Tm^3+^ (*x* = 0.4, 0.6, 0.8) phosphors fall into the nearly white light region in the CIE diagram, which makes it suitable for the fabrication of white light emitting LEDs (Shi et al., 2014; Seo et al., 2017). Therefore, the white light can be effectively adjusted by changing the Er^3+^ doping concentration.

**Figure 4:**
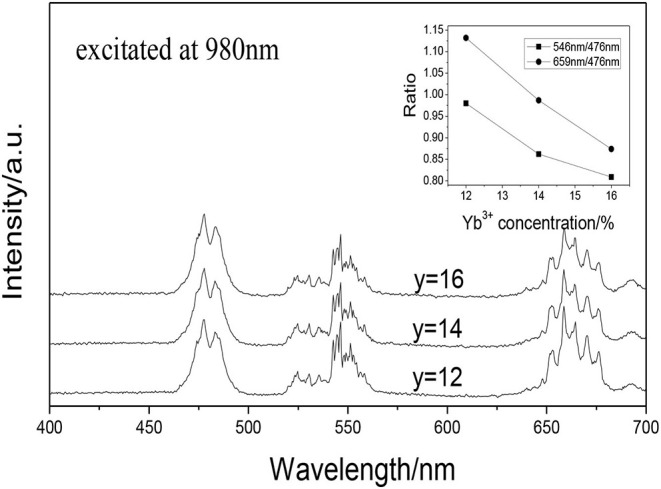
UCPL spectra of the GdAlO_3_: 0.6%Er^3+^, y%Yb^3+^, 1%Tm^3+^ phosphors (inset is the dependence of the 546 nm/476 nm and 659 nm/476 nm intensity ratio on Yb^3+^ concentration).

Figure 4 shows the UCPL spectra of the GdAlO_3_: 0.6%Er^3+^, y%Yb^3+^, 1%Tm^3+^ (*y* = 12, 14, 16) phosphors and the dependence of the 546 nm/476 nm and 659 nm/476 nm intensity ratio on Yb^3+^ concentration. It can be seen that the relative intensity of red to blue emission and the relative intensity of green to blue emission shows a downward trend with the increase of Yb^3+^ doping concentration.

**Figure 5:**
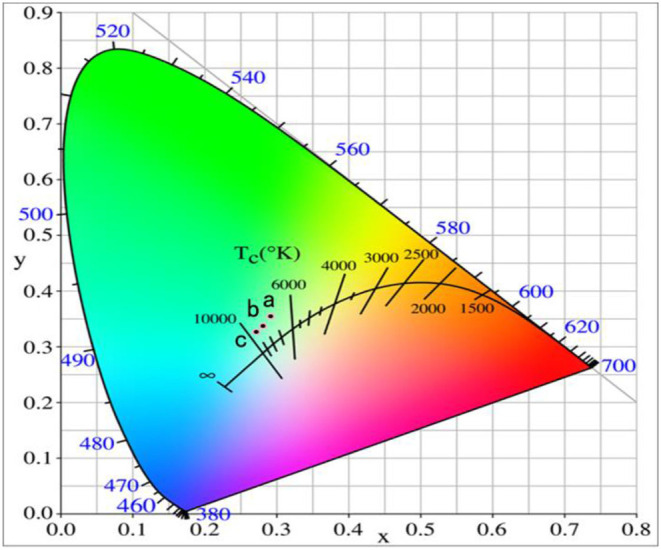
Chromaticity coordinate of the GdAlO_3_: 0.6%Er^3+^, y%Yb^3+^, 1%Tm^3+^ phosphors under 980 nm excitation (a:*y* = 12, b:*y* = 14, c:*y* = 16).

Figure 5 represents the CIE diagram of the phosphors with different Yb^3+^ doping concentrations. The CIE are (0.2926, 0.3542), (0.2811, 0.3373), and (0.2725, 0.3272) as shown in the points a, b, and c when the Yb^3+^ doping concentration was 0.12, 0.14, and 0.16, respectively. The CIE moves to the blue light direction with the increase of the Yb^3+^ doping concentration, but the moving range was not as large as that of the Er^3+^ doping, and the white light can be adjusted slightly by changing the Yb^3+^ doping concentration.

### The Effect of the Calcining Temperatures on the Tunable UC White Emissions

**Figure 6:**
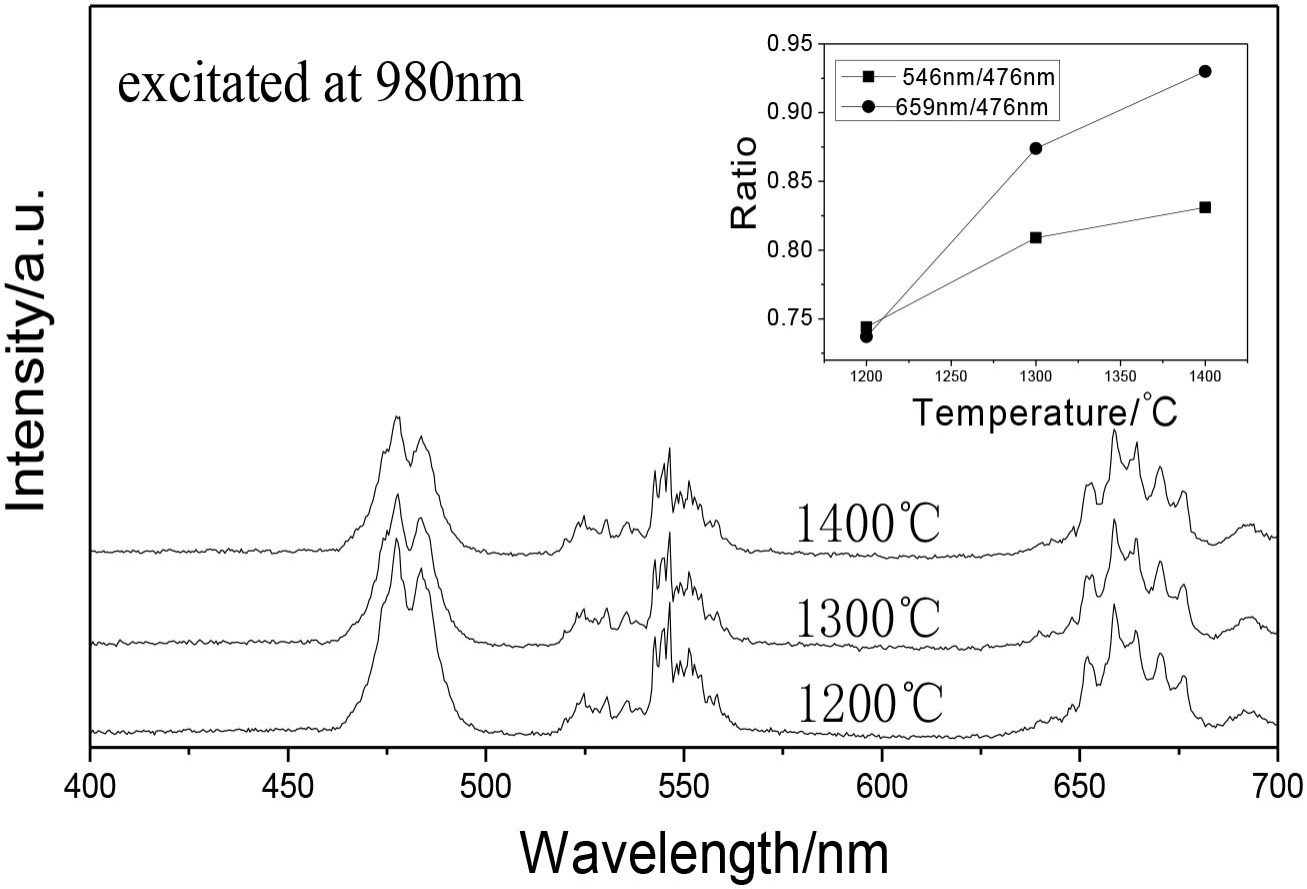
UCPL spectra of the GdAlO_3_: 0.6%Er^3+^, 16%Yb^3+^, 1%Tm^3+^ phosphors prepared by calcining the precipitate at different temperatures (inset is the dependence of the 546 nm/476 nm and 659 nm/476 nm intensity ratio on calcination temperature).

**Figure 7:**
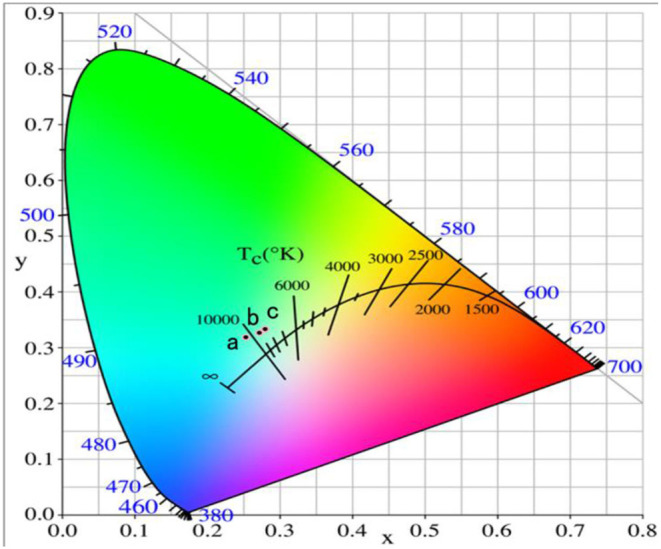
Chromaticity coordinate of the GdAlO_3_: 0.6%Er^3+^, 16%Yb^3+^, 1%Tm^3+^ phosphors prepared by calcining the precipitate at different temperatures (a:1,200°C, b:13,00°C, c:1,400°C).

It can be seen that GAP phosphor can exist stably by calcining the precipitate from 1,200 to 1,400°C in Figure 1. Figure 6 shows the UCPL spectra of the GdAlO_3_: 0.6%Er^3+^, 16%Yb^3+^, 1%Tm^3+^ phosphors prepared by calcining the precipitate at different temperatures and the dependence of the 546 nm/476 nm and 659 nm/476 nm intensity ratios on calcination temperatures at 1,200, 1,300, and 1,400°C. It can be seen that the blue emission intensity decreases, while the ratios of red to blue emission and green to blue emission intensity increase with the increase of the calcination temperature. The corresponding coordinates were (0.2596, 0.3189), (0.2725, 0.3272), and (0.2796, 0.3328) as shown in points a, b, and c, respectively, when the calcination temperatures were 1,200, 1,300, and 1,400°C from Figure 7. Moreover, all the points are located in the white light area, and the color coordinates move to the red and green emission direction. The moving range was less than that of the Er^3+^ and Yb^3+^ doping. The UC white light can be further adjusted on the basis of the Er^3+^ and Yb^3+^ doping concentration by changing the calcination temperature.

### The Effect of the Excitation Laser Powers on the Tunable UC White Emissions

**Figure 8:**
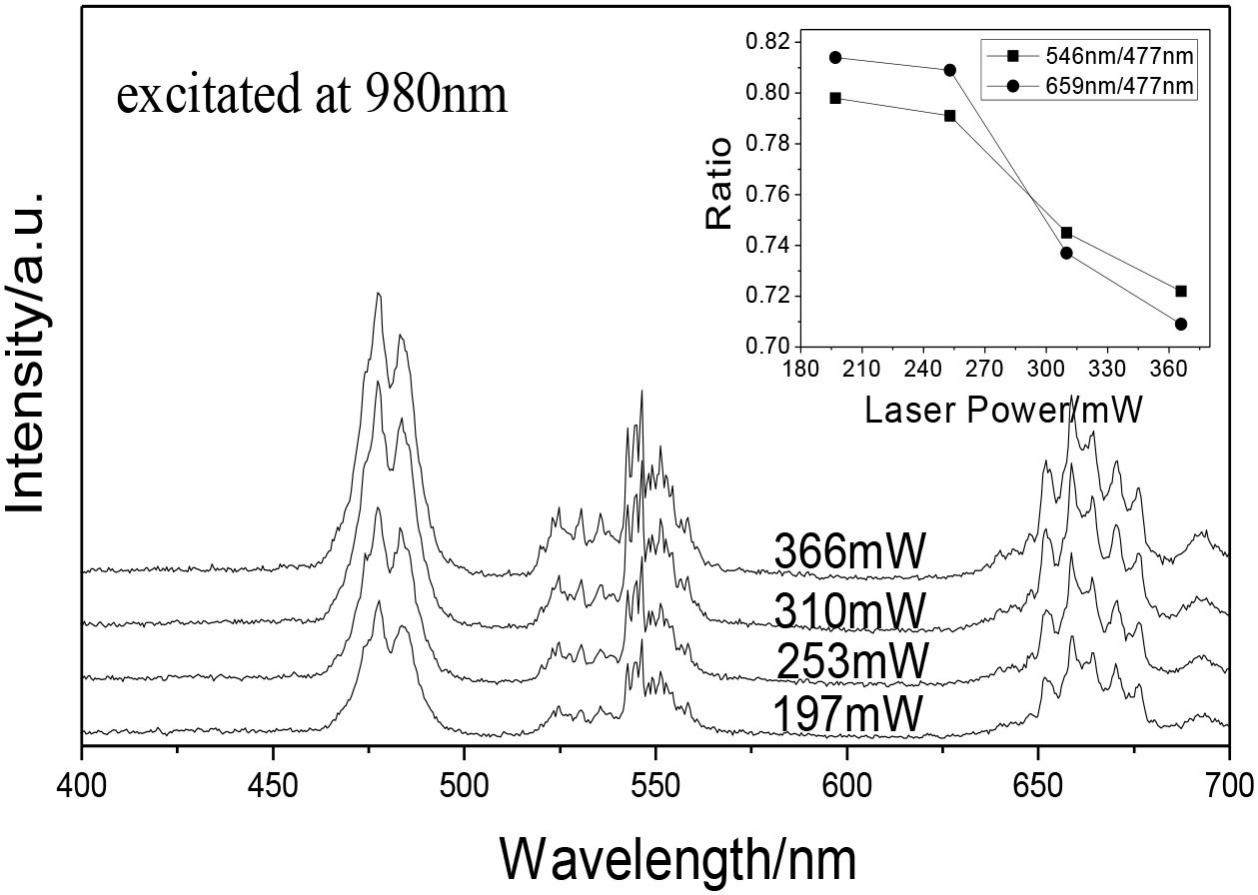
UCPL spectra of the GdAlO_3_: 0.6%Er^3+^, 16%Yb^3+^, 1%Tm^3+^ phosphor under excitation at 980 nm with different laser powers (inset is the dependence of the 546 nm/476 nm and 659 nm/476 nm intensity ratio on laser power).

**Figure 9:**
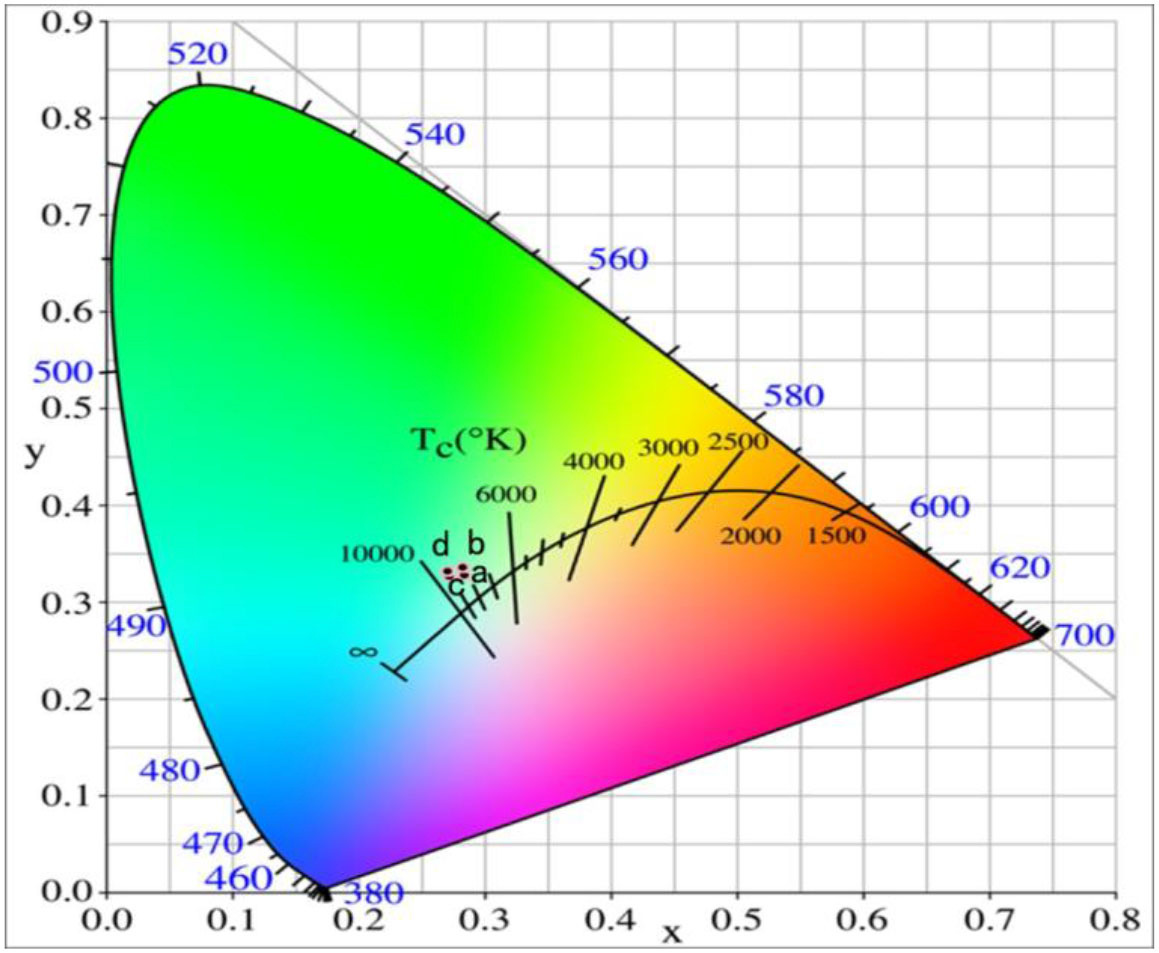
Chromaticity coordinates of the GdAlO_3_: 0.6%Er^3+^, 16%Yb^3+^, 1%Tm^3+^ phosphor under excitation at 980 nm with different laser powers (a:197 mW, b:253 mW, c:310 mW, d:366 mW).

Figure 8 shows the UCPL spectra of the GdAlO_3_: 0.6%Er^3+^, 16%Yb^3+^, 1%Tm^3+^ phosphor and the dependence of the 546 nm/476 nm and 659 nm/476 nm intensity ratios under excitation at 980 nm with different laser powers. It can be seen that the intensity of each emission peak was improved with the increase of laser power, but the relative intensities of red to blue emission and green to blue emission decrease obviously. Figure 9 presents the chromaticity coordinate of the GdAlO_3_: 0.6%Er^3+^, 16%Yb^3+^, 1%Tm^3+^ phosphor under excitation at 980 nm with different laser powers at 197 mW, 253 mW, 310 mW, and 366 mW, the CIE are (0.2846, 0.3277), (0.2825, 0.3360), (0.2725, 0.3272), and (0.2707, 0.3320) as shown in the points a, b, c, and d, respectively. Moreover, the movement of color coordinates was very small and all the points fall near the white light area.

### The Effect of Doping Li^+^ on the Tunable UC White Emissions

**Figure 10:**
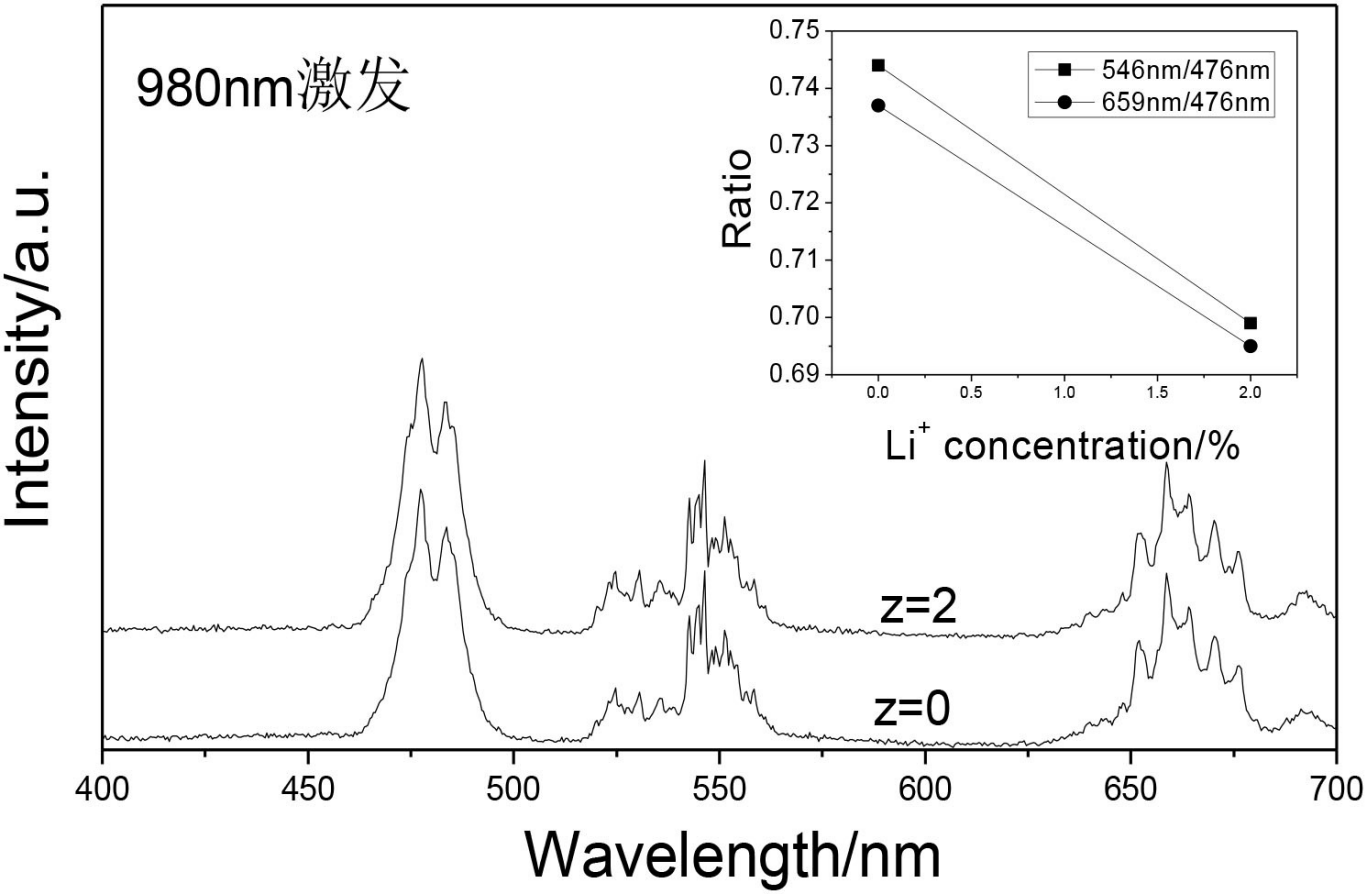
UCPL spectra of the GdAlO_3_:0.6%Er^3+^, 16%Yb^3+^, 1%Tm^3+^, z% Li^+^ phosphors (inset is the dependence of the 546 nm/476 nm and 659 nm/476 nm intensity ratio).

**Figure 11:**
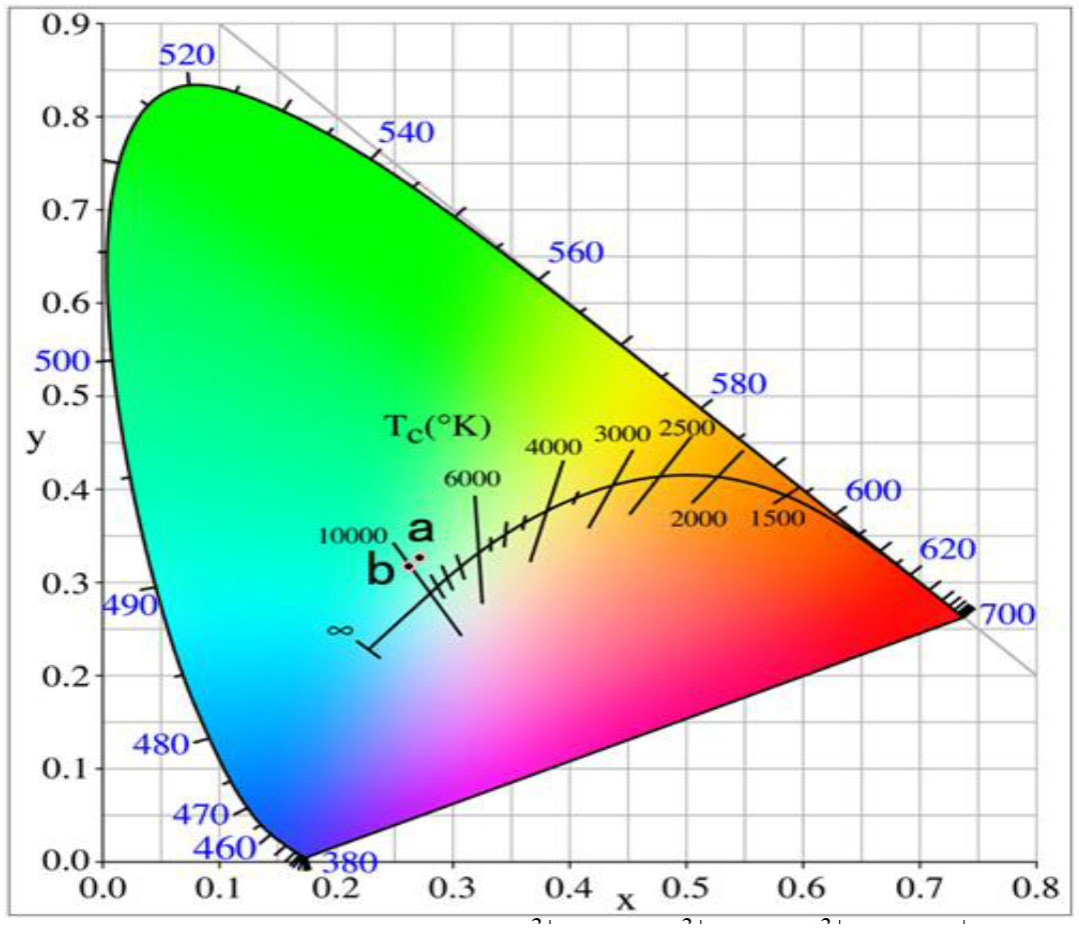
Chromaticity coordinates of the GdAlO_3_:0.6%Er^3+^, 16%Yb^3+^, 1%Tm^3+^, z% Li^+^ phosphors (1:*z* = 0; 2:*z* = 2).

Figure 10 shows the UCPL spectra of the GdAlO_3_:0.6%Er^3+^, 16%Yb^3+^, 1%Tm^3+^, z% Li^+^ phosphors and the dependence of the 546 nm/476 nm and 659 nm/476 nm intensity ratios under excitation at 980 nm. It can be seen that the blue emission intensity increases and the relative intensities of red to blue and green to blue emission decreases obviously with Li^+^ doping. The CIE was (0.2626, 0.3168), when the Li^+^ doping concentration was 0.02 at point b in Figure 11. It moves slightly to the blue direction compared with no Li^+^ doping at point a (0.2725, 0.3272). Therefore, the color coordinate position of UC white light can be adjusted slightly by Li^+^ doping.

### UCPL Mechanism

**Figure 12:**
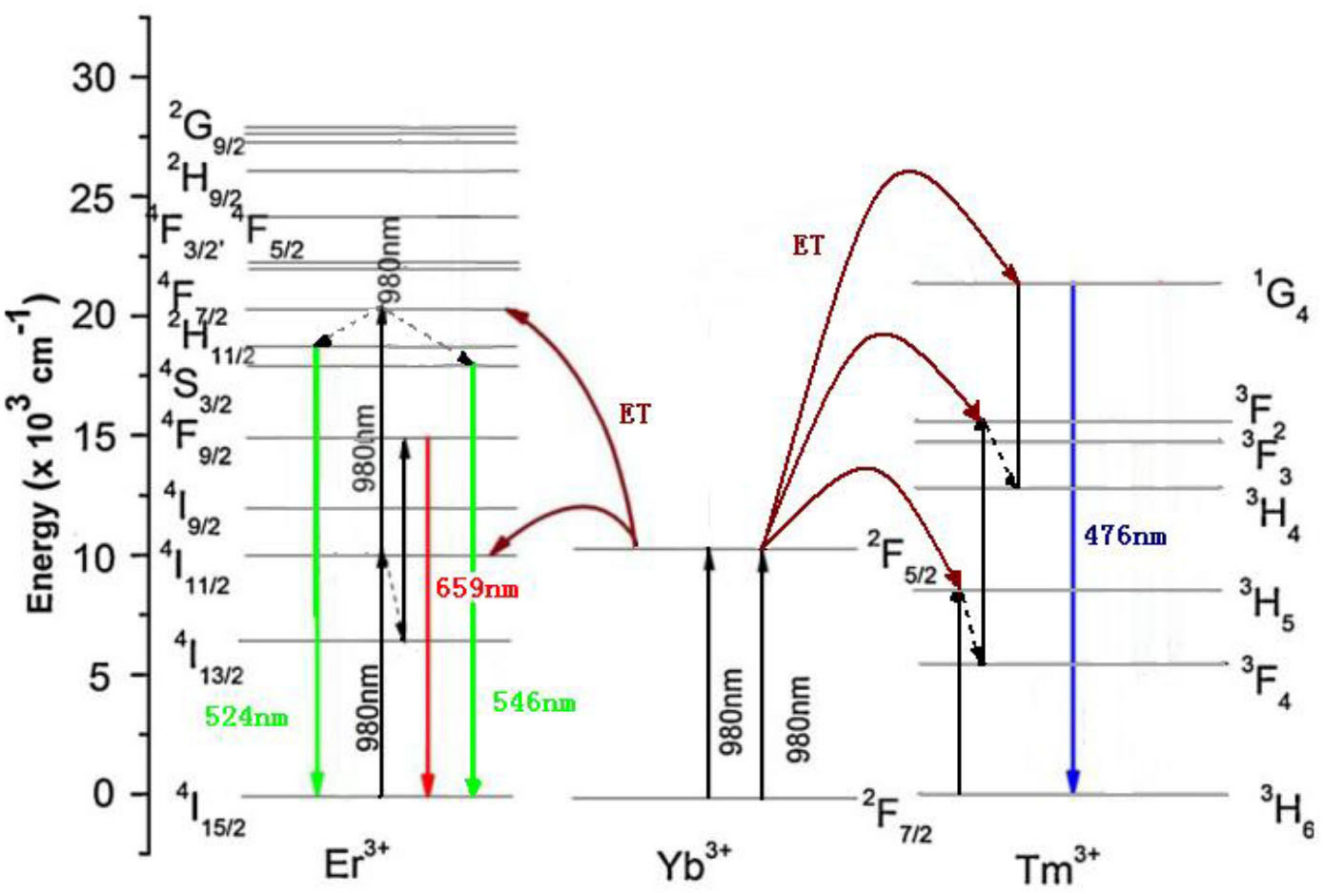
Schematic diagram of energy levels of GdAlO_3_: Er^3+^, Yb^3+^, Tm^3+^ and possible transitions under 980 nm excitation (ET: energy transfer).

The luminescence mechanism of GdAlO_3_:Er^3+^, Yb^3+^, Tm^3+^ phosphors was analyzed to reveal the UC white light chromaticity coordinate changed with different Er^3+^/Yb^3+^ concentration doping, calcining temperatures, laser powers, and Li^+^ doping. Figure 12 shows the schematic diagram of energy levels of the Er^3+^, Yb^3+^, and Tm^3+^ ions and possible transitions under 980 nm excitation. Specific processes that the green emission at 524 and 546 nm are ascribed to ^2^H_11/2_ → ^4^I_15/2_ and ^4^S_3/2_ → ^4^I_15/2_ transitions, and the red emission at 659 nm would be observed from the ^4^F_9/2_ → ^4^I_15/2_ transition all from Er^3+^, which have been discussed in our previous work (Deng and Jiang, 2018). Meanwhile, both green and red emissions are the two-photon processes. The blue emission at 476 nm would be observed from the ^1^G_4_ → ^3^H_6_ transition of Tm^3+^. The specific process was that Yb^3+^ ion, as a sensitizer first absorbed energy and transitioned from the ^2^F_7/2_ level to ^2^F_5/2_ under 980 nm irradiation, and the Tm^3+^ ion in the ground state of ^3^H_6_ was elevated to the ^3^H_5_ excited state via ET from an Yb^3+^ ion in the ^2^F_5/2_ state, then Tm^3+^ in the state of ^3^H_5_ relaxed to the ^3^F_4_ level by non-radiative relaxations. This process was followed by a second ET from another Yb^3+^ ion also in its excited state, resulting in the population of ^3^F_2_ of the Tm^3+^. After ^3^F_2_ → ^3^H_4_ fast non-radiative relaxations, the Tm^3+^ ion in the excited state of ^1^G_4_ is pumped by the third ET from an Yb^3+^ ion. Finally, the excited Tm^3+^ ion in ^1^G_4_ returned to the ^3^H_6_ ground state giving the blue emission at 476 nm, which belongs to the three-photon process. Therefore, there was a relative competition between Er^3+^ and Tm^3+^ in energy transfer from Yb^3+^ ion as the same sensitizer.

Firstly, the UC white emissions of phosphors were adjusted by changing the Er^3+^ doping concentration. It was found that the red and green emission intensity of samples increased, while that of the blue emission decreased with increased Er^3+^ doping concentration. It is not difficult to explain that the luminescence mechanism that the red and green emissions belong to Er^3+^ (^2^H_11/2_,^4^S_3/2_ → ^4^I_15/2_) and (^4^F_9/2_ → ^4^I_15/2_), while the blue emission belongs to Tm^3+^ (^1^G_4_ → ^3^H_6_). Therefore, the distance between the sensitizer Yb^3+^ and luminescent Er^3+^ became closer with the increase of the Er^3+^ doping concentration, which made the energy transfer more effective, and then the luminescent intensity of green and red emissions became higher. Meanwhile, the energy transfer between Yb^3+^ and Tm^3+^ was accordingly decreased, and to some extent, the blue emission intensity was reduced. The UC white light can be further adjusted by changing the doping concentration of sensitizer Yb^3+^. It was found that the intensity ratio of red to blue and the green to blue emissions decreased, and the blue emission increased obviously with an increase in the Yb^3+^ doping concentration. This was because the blue emission belongs to the three-photon process, which requires the effective energy transfer from the sensitizer Yb^3+^ to Tm^3+^ to reach the final blue emission level three times. While, red and green emissions belong to the two-photon process of Er^3+^, so it was more favorable to obtain the energy transfer from Yb^3+^ to Tm^3+^, which made the relative intensity of red to blue and green to blue emissions decrease.

Secondly, it can be reasonably explained that the intensity ratios of red to blue and green to blue emissions are enhanced by the increase of the calcination temperature of the precursor. As we know, the energy gap of non-radiative relaxation is very close to the vibrational frequency of the OH^−^ in the phosphor. The higher the concentration of OH^−^ impurity in the phosphor the higher the probability of ^4^I_11/2_ → ^4^I_13/2_, ^4^S_3/2_ → ^4^F_9/2_, ^3^F_2_ → ^3^H_4_, and ^3^H_5_ → ^3^F_4_ non-radiative transitions. The concentration of surface OH^−^ groups in the phosphor decreased gradually when the calcination temperature increased as shown in our previous work (Deng et al., 2014a). From the energy level diagram, it can be seen that the blue emission process needed three non-radiation transitions. Then, with the decrease of the OH^−^ groups on the surface of the phosphor, the probability of non-radiation transitions decreased, which mostly weakened the blue emission, resulting in the increase of relative intensity of red to blue and green to blue emissions, making the UC color coordinates move to the red and green emission direction.

Thirdly, the luminous intensity of each red, green, and blue emissions all increased with the increase of the excitation power laser. As far as we know, red and green emissions from Er^3+^ are two-photon processes, and the luminous intensity is directly proportional to the second power of the power, while the blue emission from Tm^3+^ is a three-photon process, and the luminous intensity is directly proportional to the third power of the power, so the effect of power on blue emission is more significant, resulting in the increase of blue emission intensity being larger than that of the red and green emission, then the relative intensity of red to blue emission and green to blue emission decreased, which can adjust the white light slightly again.

Finally, the UC white light was adjusted and the luminous efficiency was improved by Li^+^ doping. Li^+^ ion can work as a low melting point flux, which enhances the crystallization degree of the phosphor, meanwhile the crystal structure around the luminescent ions can be adjusted to reduce the crystal symmetry by replacing or occupying the crystal vacancy with Li^+^ doping (Zhao et al., 2013), and the color coordinate position of UC white light can be further slightly adjusted.

## Conclusions

In this paper, different methods were used to adjust the UCPL performance of GdAlO_3_:Er^3+^, Yb^3+^, Tm^3+^ phosphors to obtain ideal white light. The energy transfer between Er^3+^ and Yb^3+^ in the phosphors increased, then the ratios of red to blue emission and green to blue emission intensity were improved with the increase of Er^3+^ doping concentration, so as to change each color distribution successfully, and making the maximum shift of the CIE coordinate of phosphors. The UC white light could be further adjusted by changing the doping concentration of the sensitizer Yb^3+^, because it was good for the blue emissions of Tm^3+^ to obtain the energy transfer from Yb^3+^ than that of the red and green emissions from Er^3+^, which made the relative intensity of red to blue and green to blue emissions decrease. The ratios of red/green to blue emissions decreased with the increased calcination temperature of the precursor, while increasing the excitation laser power was conducive to the three-photon UC emission process of Tm^3+^, both enhanced the blue emission part of the UC white light, so as to slightly adjusted the UCPL. The crystal symmetry could be reduced by Li^+^ doping, which made the color coordinate position move slightly. The four different methods on the effect of phosphors UC white luminescence were researched systematically, and the UCPL mechanism was correspondingly discussed, which played an important role in adjusting the red /green /blue colors to obtain the ideal UC white emitting luminescence.

## Data Availability Statement

All datasets generated for this study are included in the article/supplementary material.

## Author Contributions

TD was in charge of designing the experiments and writing the manuscript. XJ performed experiments. TD and QZ were in charge of revising the manuscript. All authors contributed to the article and approved the submitted version.

## Conflict of Interest

The authors declare that the research was conducted in the absence of any commercial or financial relationships that could be construed as a potential conflict of interest.
